# Neural Representation of Ambiguous Visual Objects in the Inferior Temporal Cortex

**DOI:** 10.1371/journal.pone.0076856

**Published:** 2013-10-03

**Authors:** Nazli Emadi, Hossein Esteky

**Affiliations:** 1 School of Cognitive Sciences, Institute for Research in Fundamental Sciences (IPM), Tehran, Iran; 2 Research Center for Brain and Cognition, School of Medicine, University of Shahid Beheshti, Tehran, Iran; 3 Howard Hughes Medical Institute and Department of Neurobiology, School of Medicine, Stanford University, Stanford, California, United States of America; Australian National University, Australia

## Abstract

Inferior temporal (IT) cortex as the final stage of the ventral visual pathway is involved in visual object recognition. In our everyday life we need to recognize visual objects that are degraded by noise. Psychophysical studies have shown that the accuracy and speed of the object recognition decreases as the amount of visual noise increases. However, the neural representation of ambiguous visual objects and the underlying neural mechanisms of such changes in the behavior are not known. Here, by recording the neuronal spiking activity of macaque monkeys’ IT we explored the relationship between stimulus ambiguity and the IT neural activity. We found smaller amplitude, later onset, earlier offset and shorter duration of the response as visual ambiguity increased. All of these modulations were gradual and correlated with the level of stimulus ambiguity. We found that while category selectivity of IT neurons decreased with noise, it was preserved for a large extent of visual ambiguity. This noise tolerance for category selectivity in IT was lost at 60% noise level. Interestingly, while the response of the IT neurons to visual stimuli at 60% noise level was significantly larger than their baseline activity and full (100%) noise, it was not category selective anymore. The latter finding shows a neural representation that signals the presence of visual stimulus without signaling what it is. In general these findings, in the context of a drift diffusion model, explain the neural mechanisms of perceptual accuracy and speed changes in the process of recognizing ambiguous objects.

## Introduction

Inferior temporal (IT) cortex, as the last stage in the ventral visual pathway, contains neurons that selectively respond to complex visual objects such as faces and bodies [1-4]. Activity of the category selective neural clusters in IT has been shown to be causally linked with perceptual decision making [5].

Visual objects in natural scenes appear in different sizes, orientations, colors, contrast, views and positions. While the level of the tolerance of IT neurons to such variations in the visual stimuli has been extensively explored [3,4,6-12], the effect of ‘ambiguity’ of the visual objects on IT neural responses is not clear yet. In our everyday life there are many situations that visual stimuli are degraded and stimulus visibility is poor. Driving in heavy rain or snow is an example of a situation where we need to recognize degraded visual objects such as pedestrians through windshield covered with snow or raindrops. Often, recognition of these ambiguous objects needs to be done as fast and accurately as possible to take the appropriate action. Psychophysical studies have shown that the accuracy and speed of the object recognition decreases as the stimulus ambiguity increases [5,13,14].

Our aim was to study the neural representation of ambiguous visual objects and the underlying neural mechanisms of such behavioral changes. We recorded the IT neural spiking activities of two macaque monkeys while passively viewing ambiguous body and object images. Stimuli were degraded by various levels of noise. We have previously shown the presence of neural clusters in IT that respond selectively to human and animal bodies [2]. Here we analyzed the body category selective units of IT to address four questions: 1) What is the relationship between the level of stimulus ambiguity and the response amplitude? 2) What is the effect of noise on the temporal dynamic of the neural responses? 3) What is the relationship between the category selectivity and various levels of noise? 4) What is the neural mechanism of decreased accuracy and speed in recognizing ambiguous stimuli? To answer the last question we present our results in the context of a drift diffusion model of decision making. Our findings shed light on the neural representation of ambiguous objects and the neural mechanisms of decreased accuracy and speed of object recognition in noisy conditions.

## Methods

### Subjects and Ethics Statement

Two male adult macaque monkeys were used in this study. Before training, the monkeys were prepared with head restraints and recording chambers implanted stereotaxically on the dorsal surface of their skull. Implantation was performed under aseptic conditions while monkeys were anesthetized with sodium pentobarbital. All experimental procedures were in accordance with the National Institutes of Health guide for the care and use of laboratory animals. They were also approved by the animal care and use committee of Institute for Research in Fundamental Sciences (04-11-64122008). Some ethical standards incorporated into our routine laboratory procedures include housing the primates in a large space with sunshine, providing them with a psychological enriched environment (TV and toys), frequent contact with other animals (visual, auditory, touch and grooming) and pharmacological amelioration of pain associated with surgeries. The monkeys received food twice a day including fresh fruits, nuts, vegetables and special biscuits. None of the animals used in this study were sacrificed.

### Stimuli

**Figure 1:**
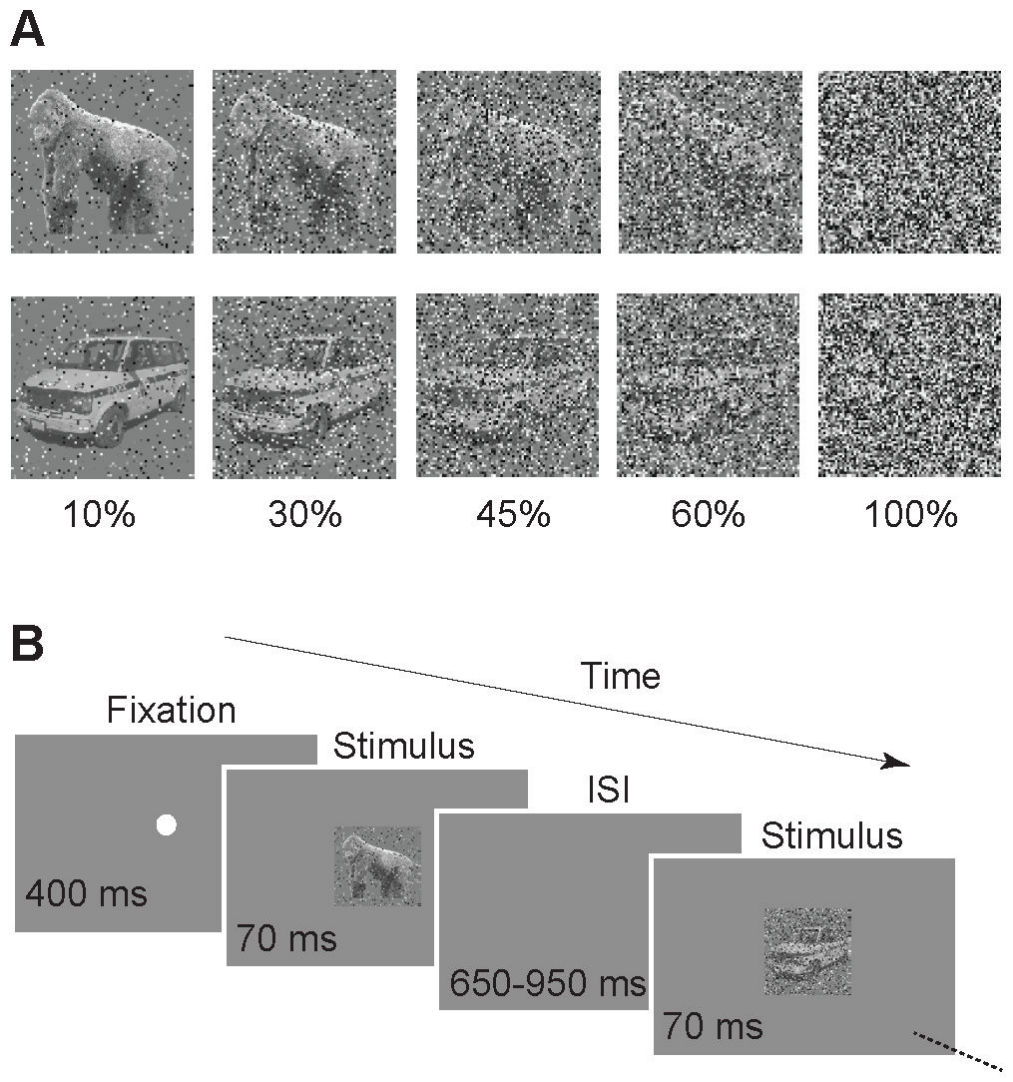
Stimuli and task. A. Exemplar body (monkey body) and object (car) stimuli in different levels of noise. Numbers below the images show percent of the noise for each column of images. B. Sequence of task events. Two macaque monkeys were trained to perform a passive viewing task. The presentation of the stimulus sequence started after the monkey maintained fixation for 400 ms on a small white fixation point at the center of the screen. Images from two different categories (body and object) were presented randomly for 70 ms with a variable (650 to 950 ms) inter-stimulus interval (ISI). The monkeys’ task was to keep their gaze fixed on the center of the screen. They were rewarded every 1.5 to 2 seconds for maintaining the fixation.

The stimuli were 7° x7° in size grayscale photographs of body (including human, monkey and quadruped subcategories) and object categories (including aircraft, car and chair subcategories). There were 90 images in each category (30 images per subcategory, Figure S1). Body images had no facial features. Each stimulus was presented in four different noise levels. Each noise level was generated by assigning a uniformly distributed grayscale value to a random selection of X% of image pixels, where X was the absolute noise level and had one of the values of 10, 30, 45 or 60. These 720 noisy stimuli [(2 categories) x (90 stimuli in each category) x (4 noise levels)] and 90 full noise images (100% noise) were randomly presented to the monkeys without repetition. Figure 1A shows two exemplar images in different noise levels. The noise pattern was fixed for one stimulus but different among stimuli. The stimuli were presented on a 19 inch CRT computer monitor placed 57 cm in front of the monkey seated in a primate chair.

Among different methods of degrading the visual information (such as phase-scrambling, morphing, etc.), we chose the high-frequency "salt & pepper noise” for our study. Large receptive fields of the IT cells and the low-pass filtering process make these cells more robust to such a high-frequency noise, compared to the smaller receptive fields of cells in lower visual areas such as V1. Taking advantage of this property of the IT cells and by using different noise levels, we studied the IT neural responses in different levels of ambiguity.

### Task

Monkeys were trained to perform a passive viewing task (Figure 1B). Following 400 ms of fixation on a white fixation point at the center of the screen, a randomly selected sequence of images were presented to the monkey. Each image was presented for 70 ms with a variable inter-stimulus interval (650 to 950 ms). The monkey was rewarded with a drop of apple juice every 1.5–2 seconds as long as its gaze was fixated within a 2.4° x2.4° invisible fixation window at the center of the screen. The eye position was measured by an infra-red eye-tracking system. The sequence of images stopped when the monkey broke the gaze fixation and the fixation point reappeared after 1500 ms of blank interval. Stimuli in the broken trials were presented again later. Recording sessions in which monkeys successfully completed at least half of the trials are included in the analysis. Median number of trials per category was 360 (mean±sem: body category=352±13, object category=353±13).

### Recording

Craniotomy was performed to record from the inferior temporal cortex of the monkeys. The recording positions were defined by the stereotactic measurements and magnetic resonance images (MRIs) acquired prior to surgery. Subdivisions of the IT cortex were defined using the location of cortical sulci as described by Tanaka and colleagues [15-17]. Recordings were made on an evenly spaced grid, with 1-mm intervals between penetrations over a wide region of the lower bank of superior temporal sulcus (STS) and TE cortical areas (12 to 18 mm and 13 to 20 mm anterior to interauricular line in monkey1 and monkey2, respectively). During each recording session, a single tungsten electrode (FHC, 0.5 to 1 MΩ) was inserted into the IT cortex. The electrode was advanced with an Evarts-type manipulator (Narishige, Japan) from the dorsal surface of the brain through a stainless steel guide tube inserted into the brain down to 10-15 mm above the recording sites. Neural activity of multi-units (MU) in the inferior temporal cortex was recorded extracellularly, while monkeys were performing the task. To separate the spiking activity from noise we set a threshold in each recording session depending on the signal quality and the amplitude of the spikes relative to the baseline noise. Each recorded MU was the superimposed activity of several neurons around the electrode tip. A total of 66 visually responsive MUs were recorded from two monkeys (41 from monkey1 and 25 from monkey2). Visual responsiveness was defined as significantly larger evoked responses compared to the baseline activity occurring in at least one of the noise levels in any of the sliding 50-ms windows from 100 to 300 ms after the stimulus onset (t-test, alpha= 0.01). The baseline activity was measured during -50 to 0 ms relative to the stimulus onset. We explored the MU activity (MUA) because of several reasons:

First, several studies have shown that SUs and MUs in different cortical areas such as V1 and V4 behave in a similar fashion [18-20]. In V1, the response onset latency and the timing of the modulations in the response caused by the context or attention have been shown to be similar in MUAs and SUAs during a figure-ground task [19]. . We also know that MUs are even more informative than SUs for movement prediction in the motor cortex [21]. This large amount of information retained in the superimposed activity of multiple neurons suggests that the response properties of adjacent neurons are consistent and neighboring cells process similar information [19,21].

Second, Previous studies have shown that cells with similar selectivity are clustered in columns in the IT cortex [22-24]. It has also been shown that the object selectivity of a given cell in an active optical imaging spot is similar to that of the averaged cellular activity within the spot [25]. Therefore, recorded MUs in the IT cortex consist of a group of homogenous SUs with similar response properties, rather than reflecting the activity of heterogeneous single cells with larger response amplitudes. Considering the similarity of the response properties of nearby neurons it could be advantageous to collect the activity of a pool of neurons to increase the signal/noise ratio.

Third, MU recordings have some technical advantages over SU recordings. They do not require spike isolation and are more stable over time [19,26]. A concern about SU recordings is that the neurons with large action potentials are more easily and reliably isolated as a SU, creating a bias towards large neurons [19]. Furthermore, reliable isolation of SUs during a recording session is not always possible.

Forth, the goal of our study was to examine the modulations of different response properties of the IT cells by stimulus ambiguity. We added different levels of visual noise to our images to explore a full range of visual ambiguity. We predicted a significant decrease in the evoked response of IT cells especially in high noise levels. So in our study in order to obtain a more reliable signal to noise ratio we focused on MUA. We believe that especially for exploring the pattern of modulation of different response properties in time (such as response onset latency, offset latency and duration, and also SI modulation in time), the higher reliability of MUA is a clear advantage in this study.

However, the advantages of analyzing MUA do not imply that they are necessarily used by the brain. The MUA could be considered as a tool to better understand the brain physiology [21], just like LFPs [27] or functional MRI [28].

### Data Analysis

Based on the similarity of the results in monkey1 and monkey2, data from two monkeys were combined in all of the analyses.

### Analysis of the Amplitude of the Evoked Response

The window used for the analysis of the evoked response was 100 ms to 300 ms after stimulus onset, unless otherwise mentioned. Changing this time to other windows (e.g. 70 to 300 ms after stimulus onset) did not change any of the main results.

### Analysis of the Selectivity Index (SI)

The degree of category selectivity of each unit for body versus object images was measured by: 

$$SI=\frac{\mu (Β)-\mu (Ο)}{\mu (Β)+\mu (Ο)}\times 100$$

**Figure 2:**
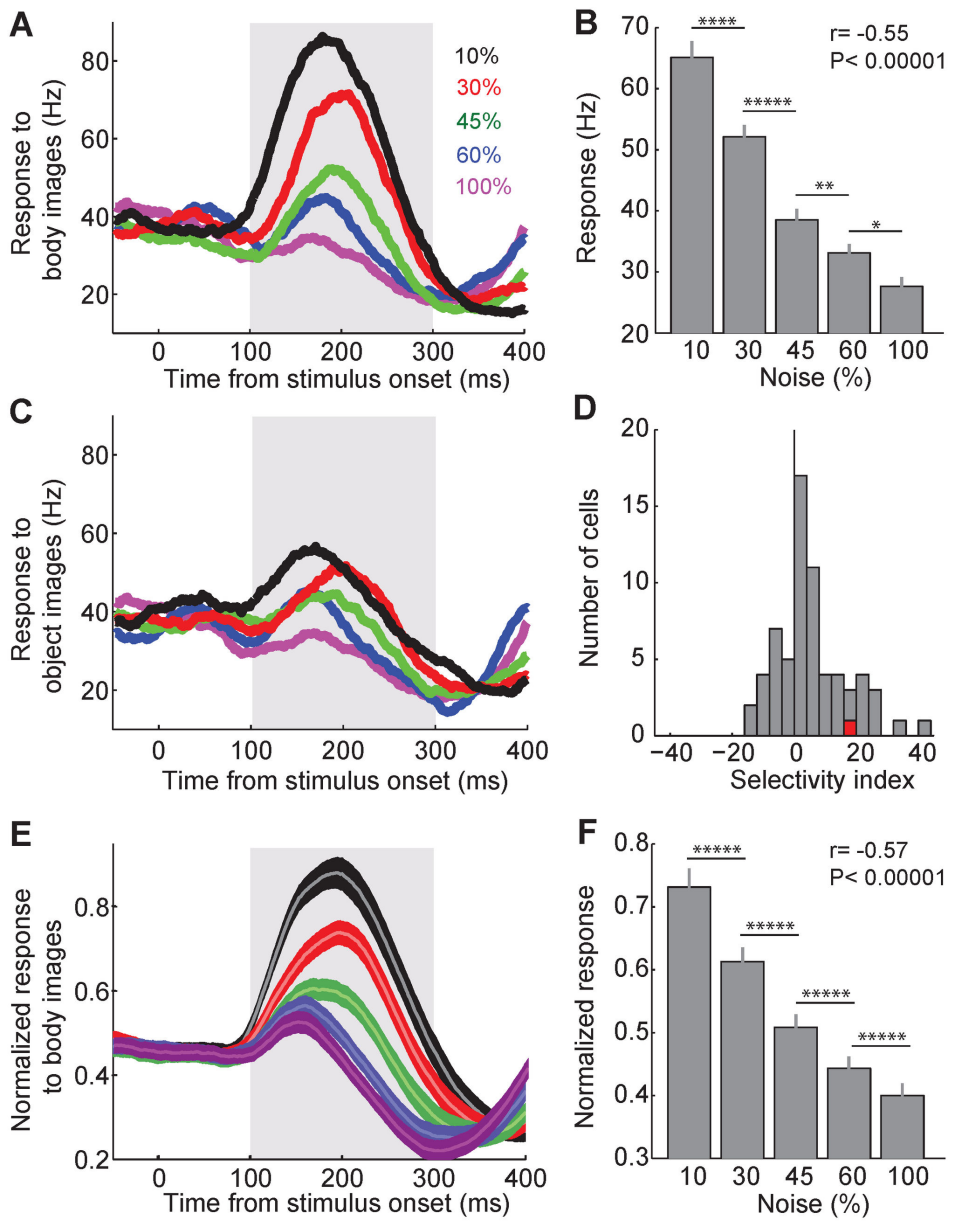
Response amplitude in different noise levels. A. The response of an exemplar unit (U10) to body images in different noise levels (black: 10%, red: 30%, green: 45%, blue: 60% and magenta: 100%). Here and in all other plots of temporal pattern of the events, responses of units were measured in 1-ms bins and smoothed by convolving with a 30-ms Gaussian kernel. The gray box represents the period of evoked activity used for the further analysis. B. Mean response of the exemplar unit in A (U10) to body images with different levels of ambiguity, during 100 to 300 ms after stimulus onset. Error bars denote s.e.m. across different trials. Stars show the p-values of the t-test between pairs of noise levels (*: P<0.05; **: P<0.01; ***: P<0.001; ****: P<0.0001; *****: P<0.00001). Inset r and P show the correlation coefficient and its p-value for the Pearson correlation analysis between responses and noise levels. C. The response of the exemplar unit (U10) to object images. Conventions as in A. D. Distribution of the body selectivity index (see Methods) for all of the recorded units. Red data point shows the exemplar unit (U10). E. Averaged response of all units to body images with different levels of noise. For normalization, peak response of each unit was measured before smoothing. Then smoothed responses of each unit in different noise levels were normalized to the peak response of that unit. Finally, normalized responses of different units in each noise level were averaged. Shaded area shows s.e.m. across different units. Conventions as in A. F. Mean normalized response of all units to body images in different levels of noise, during 100 to 300 ms after stimulus onset. Error bars here and in other figures denote s.e.m. across different units. Conventions as in B.

μ(B) and μ(O) were the mean evoked response of each unit to body and object images in 10% noise level, respectively. This index could vary from -100 (absolute object category selectivity) to 100 (absolute body category selectivity). Units with SI values larger than zero were considered as ‘body-selective’. With this definition there were 48 body-selective units in our data set (Figure 2D). The SI values of our units could not be directly compared to the selective responses of face cells reported in other studies which used different stimulus sets. This is mainly due to the presence of 10% noise in the most visible image and the complexity of the images used as object stimuli in our study as well as the different response properties of body selective cells compared to face selective cells.

We also defined category selectivity as responses to body images with %10 noise being significantly larger than object images with 10% noise (t-test, alpha= 0.05, window: 100 to 300 ms after the stimulus onset). All of the reported results were similar if we did the analysis on the 21 category selective units selected by this definition.

### Analysis of Onset Latency, Offset Latency and Duration of the Evoked Response

In order to measure the onset, offset and duration of the evoked response, the activity of each unit was smoothed by convolving it with a 30-ms Gaussian kernel in every noise level. Smoothing was done to prevent measuring random jitters in the response as onset or offset. For each unit and noise level, the firing rate during baseline activity was compared to different windows of evoked activity (paired t-test, one-tailed, alpha= 0.01). The firing rate of single trials during -50 to 0 ms relative to the stimulus onset was defined as the baseline activity and the firing rates of single trials in sliding 50-ms windows, during 50 to 400 ms after the stimulus onset, was defined as the evoked response. The onset of the evoked response was defined as the first 50-ms window of the evoked response with values significantly larger than the baseline. The time interval between the start of this window and the stimulus onset was considered as the evoked response onset latency. The end of the evoked response was defined as the first window after the response onset with values not significantly larger than the baseline. The time interval between the start of this window and the stimulus onset was considered as the evoked response offset latency. Duration of the response was measured as offset of the response minus its onset, in each unit and noise level.

Reliable measurement of the onset and offset was not possible in several units at noisier conditions due to the smaller amplitude of the evoked response. Onset and offset latency of the response in all of the noise levels could be measured in 25 and 21 of the units, respectively. Thereby the duration of the response was calculated in 21 units. The fewer number of measurable offsets compared to the onsets is related to four MUs not returning to the baseline activity until 400 ms after the stimulus onset. Furthermore, the observed difference could be related to the low response variability at the beginning of the evoked response [29]. Therefore, there is a higher chance of obtaining statistical significance when measuring response onset compared to offset.

### Analysis of Classification

A linear ‘support vector machine’ was used to assess the neural performance. In each noise level, the evoked response of each unit to body and object images was used as an input to the classifier. In each round of classification, we randomly selected the 75% of trials of every unit in each noise level for training the classifier. The classification performance of the cell population was tested on the remaining 25% of trials. This procedure was repeated for 1000 rounds to evaluate the statistical difference in performance between conditions.

## Results

We investigated the effect of visual ambiguity on the IT neural responses by recording the spiking activities of 66 multiple units (MU) from the IT cortex of two macaque monkeys while they passively viewed noisy visual stimuli with various degrees of noise (10%, 30%, 45%, 60% and 100%) (Figure 1).

### Amplitude of the Evoked Response

To explore the effect of different levels of stimulus ambiguity on the neural response of the IT cortex, we first looked at the responses of each unit to body and object images at different levels of noise. Figure 2A shows the mean response of one exemplar unit (U10) to body images at different noise levels. As more noisy images were presented a gradual decline in the amplitude of the responses of this unit was observed. To better quantify this modulation, we measured the evoked firing rate of this unit during 100 to 300 ms after stimulus onset in each single trial (Figure 2B). Consistent with the peristimulus time histogram (PSTH) in Figure 2A, the response amplitude declined as the stimulus ambiguity increased (Pearson correlation, *r*= -0.55, *P*< 0.00001). Also its response to object images at different noise levels was smaller compared to body images (Figure 2C). We defined a selectivity index (SI) to measure this difference more directly, (see Methods). For this exemplar unit the value of SI in the lowest level of noise (10%) was 18.2 which confirmed its body selectivity. The IT cortex, as the last unimodal stage in the ventral visual pathway, has units selective to complex objects like faces and bodies [1-4,30-38]. Body selectivity in the IT cortex is reported at the level of single cell [2] and cortical patches [30,31,35,37]. We measured SI in the 66 units to document the presence of body selectivity in the activity of several neighboring cells recorded as a unit in our study. Figure 2D shows the distribution of body selectivity index in the recorded units. We identified 48 body selective units (32 from monkey1 and 16 from monkey 2) and further analyzed their spiking activities.

The averaged responses of all 48 body selective units to their preferred category (body) at different levels of noise are shown in Figure 2E. There was a decline in the response amplitude as the stimulus ambiguity increased. We noted a clear distinction of the responses to body images in different degrees of noise at the population level which indicated a similarity in the sensitivity of these units to the degradation of their preferred category. To quantify the gradual decrease in the response of units to more noisy images we measured the average of normalized responses of the units during 100 to 300 ms after stimulus onset at each noise level (Figure 2F). Consistent with the data from the exemplar unit (Figure 2B) and also the PSTH in Figure 2E, the response amplitude declined linearly as stimulus ambiguity increased (Pearson correlation; both monkeys: *r*= -0.57, *P*< 0.00001; monkey1: *r*= -0.57, *P*< 0.00001; monkey2: *r*= -0.58, *P*< 0.00001).

### Temporal Dynamic of the Evoked Response

**Figure 3:**
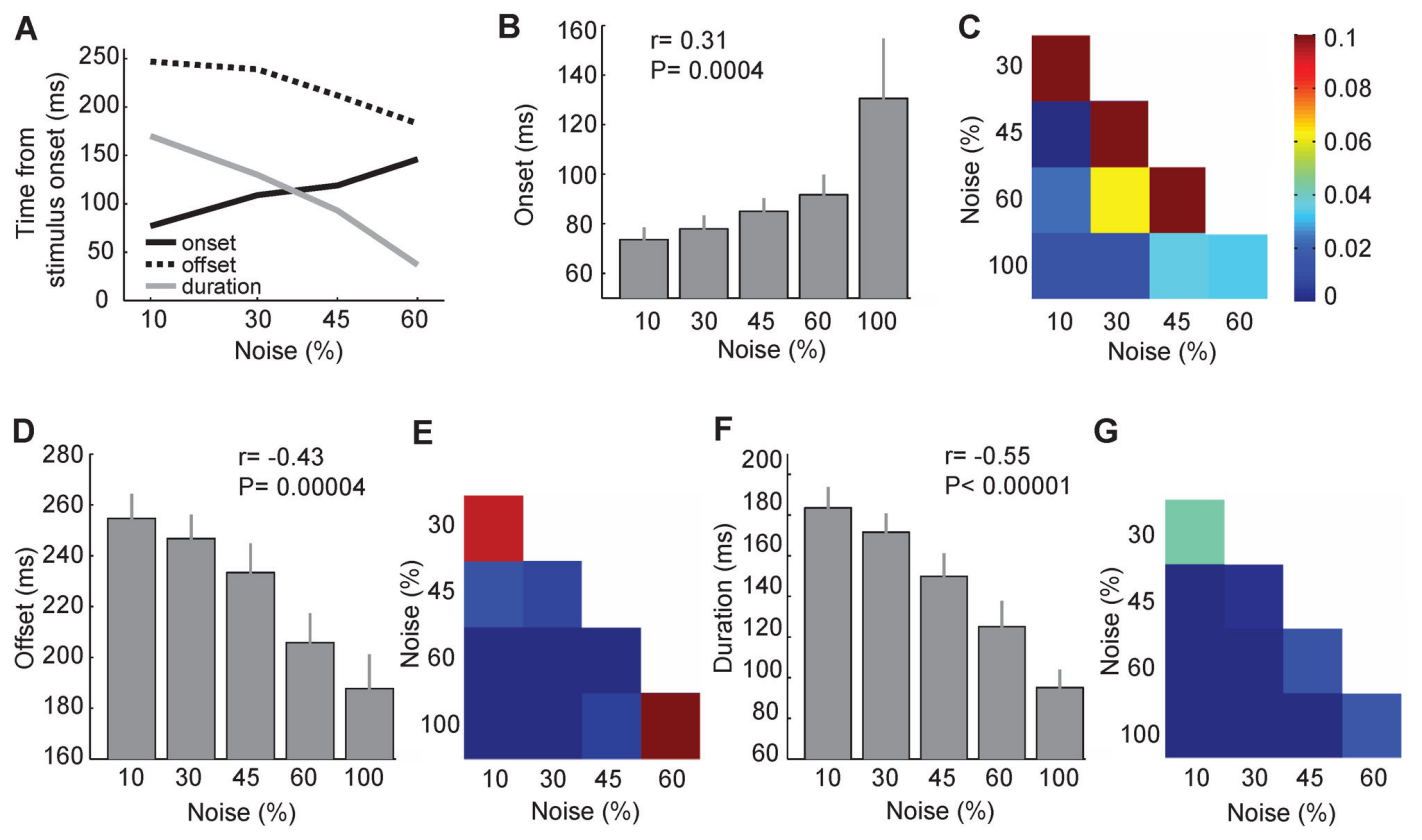
Response onset latency, offset latency and duration in different noise levels. A. Onset latency, offset latency and duration of the response of the exemplar unit (U10) to body images in different levels of ambiguity. When full noise images were presented there was no increase in the response of this unit relative to the baseline activity (Figure 2A). Therefore, onset, offset and duration were not measurable in this noise level and are not shown in this figure. B. Onset latency of the response of the units (n=25) to body images with different levels of noise. C. P-values of the comparison of onset latency values in different pairs of noise levels (t-test, paired). D. Offset latency of the response of the units (n=21) to body images with different levels of noise. E. P-values of the comparison of offset latency values in different pairs of noise levels (t-test, paired). Conventions as in C. F. Duration of the response of the units (n=21) to body images with different levels of noise. G. P-values of the comparison of response duration values in different pairs of noise levels (t-test, paired). Conventions as in C.

So far, our results showed a gradual decline in the response amplitude of the population of the body selective units as a function of noise. The amplitude of the evoked response is a common coding mechanism in different cortical areas [39,40]. Onset of the evoked response is another potential mechanism for visual stimuli coding in the IT cortex [41]. We previously demonstrated that the onset latency of the evoked response is shorter for specific categories (faces) compared to the others [41]. We predicted that the onset latency as a neural coding tool could also be affected by the level of stimulus ambiguity. Observation of the temporal pattern of responses to images in different noise levels in Figure 2 is consistent with this idea. In Figure 2A the onset and offset of the response of the body selective unit to its preferred category was found to be different among noise levels. To better quantify this effect, we measured the onset, offset and duration of the response of this unit (U10) to body images in different levels of stimulus ambiguity (see Methods). The evoked response of this unit started later and decayed earlier as stimulus ambiguity increased (Figure 3A). As a result of these modulations, the duration of the response was shorter for more ambiguous images (Figure 3A).

Based on these findings and also the temporal pattern of the population response in Figure 2E, we expected to see similar results in other body selective units. Figure 3B shows the onset of the response of body units to body images with different levels of ambiguity. Response onset was earlier for less noisy images (Figure 3B; Pearson correlation; both monkeys: *r*= 0.31, *P*= 0.0004; monkey1: *r*= 0.31, *P*= 0.0046; monkey2: *r*= 0.34, *P*= 0.03; see also Figure 3C). We found a larger variability of the onset latency among body units at 100% noise level indicating possible increased within unit variability of the onset latency in this condition.

We then tested the correlation between the response amplitude and response onset latency across all noise levels (*r*= -0.38, *P*<0.00001). We also measured the onset latency of the response to object images in different noise levels (Pearson correlation, *r*= 0.22, *P*= 0.02). Similar to the body images, we found a significant negative correlation between the response amplitude and the onset latency (*r*= -0.46, *P*<0.00001). One concern with respect to this finding might be that the shorter onset latency in less noisy conditions was simply the result of a larger response amplitude in these conditions [17]. We addressed this issue using two-way ANOVA in which image categories and noise levels were the two factors to compare the onset latency of the responses to body and object images at different noise levels. The results showed that the onset latency was different across noise levels (*P*= 0.006), while it remained unchanged between two categories (*P*= 0.24). We also compared the amplitude of the response to body and object images at different noise levels using two-way ANOVA. We found that the response amplitude was significantly different both across noise levels (*P*< 0.00001) and between categories (*P*< 0.00001, larger response amplitude for bodies). Collectively these results suggest that while the amplitude of the response to bodies was larger than the response to objects at different levels of noise, the onset of the responses were not different at any given level of noise. Therefore, if the observed difference in the response onset latency across noises (Figure 3B) was simply the result of the difference in the response amplitude, it should have been also different between body and object images at each noise level. This analysis confirms that the modulations in the onset latency could not be explained by the differences in the response amplitude.

We also measured the offset latency and duration of the response which both decreased as more ambiguous stimuli were presented (offset: Figure 3D; Pearson correlation; both monkeys: *r*= -0.43, *P*= 0.00004; monkey1: *r*= -0.45, *P*= 0.0001; monkey2: *r*= -0.47, *P*= 0.0095; see also Figure 3E) (duration: Figure 3F; Pearson correlation; both monkeys: *r*= -0.55, *P*< 0.00001; monkey1: *r*= -0.57, *P*< 0.00001; monkey2: *r*= -0.57, *P*= 0.0011; see also Figure 3G). These findings suggest that the increased duration of the evoked response to more visible images is the result of an earlier rise and a later fall in the response.

### Selectivity of the Evoked Response

**Figure 4:**
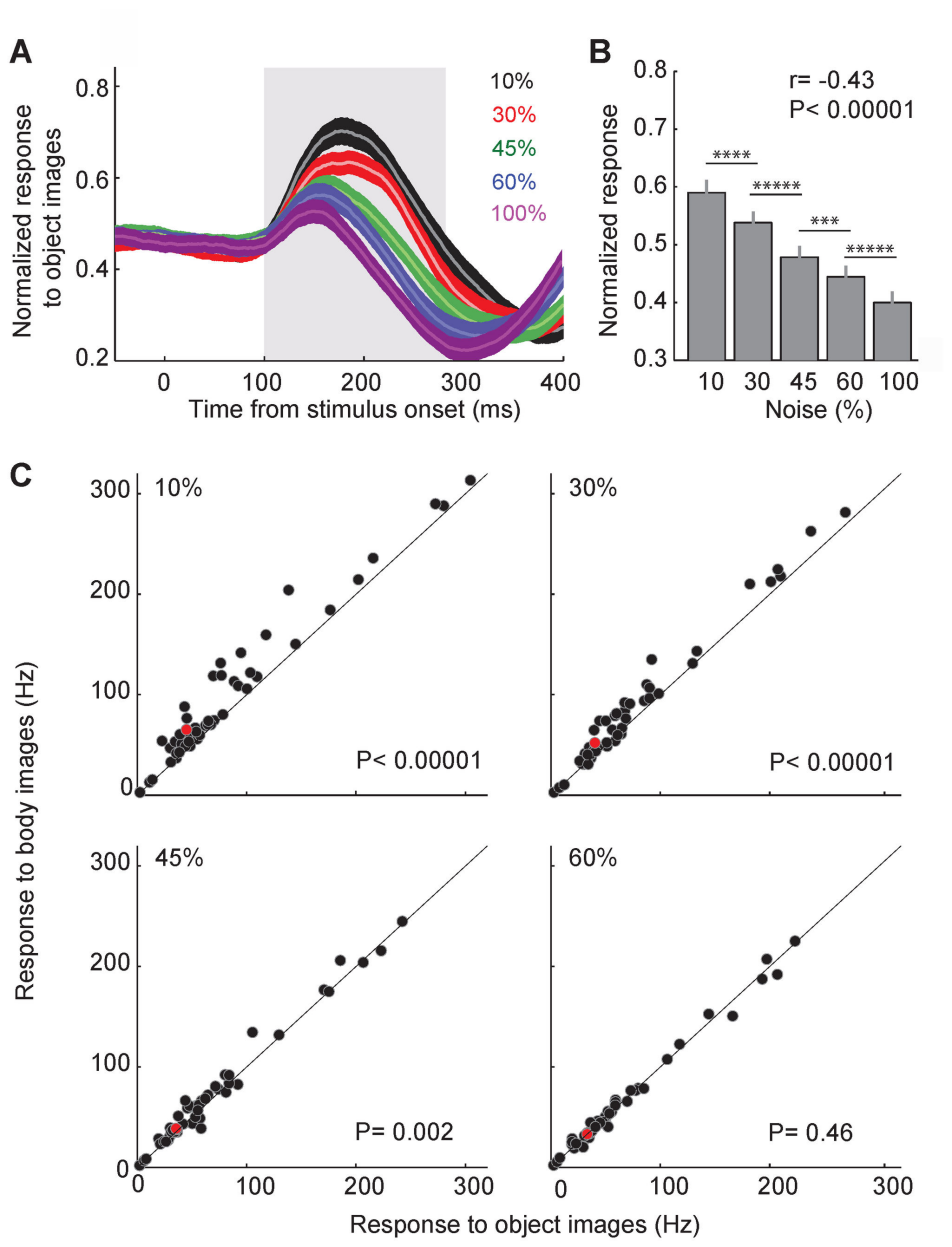
Response to body vs. object images in different noise levels. A. Averaged response of all units to object images with different levels of noise. Normalization was done as described in Figure 2E. The gray box represents the period of evoked activity used for the further analysis. B. Mean response of all units to object images in different levels of noise, during 100 to 300 ms after stimulus onset. Conventions as in Figure 2B. C. Response of all body selective units (n=48) to body and object images in different noise levels during 100 to 300 ms after stimulus onset. Each data point shows the mean response of one unit. The red data point shows the exemplar unit (U10). Full noise (100%) is not shown in this figure because there is no category information in full noise images. The inset p-values show the results of paired t-ttest between responses to body and object images.

Exploring the noise-related modulation of the responses to the preferred category helps to understand the cortical sensory processing. However, for a better understanding of the effect of noise on object recognition it is essential to compare noise-related modulations of the responses to the preferred versus non-preferred category (category selectivity). The responses of the body selective units to the non-preferred category (object images) are shown in Figure 4A. These responses were smaller than the responses to body images (Figure 2E) and declined as the noise level increased (Figure 4B; Pearson correlation; both monkeys: *r*= -0.42, *P*< 0.00001; monkey1: *r*= -0.43, *P*< 0.00001; monkey2: *r*= -0.42, *P*= 0.0001).

**Figure 5:**
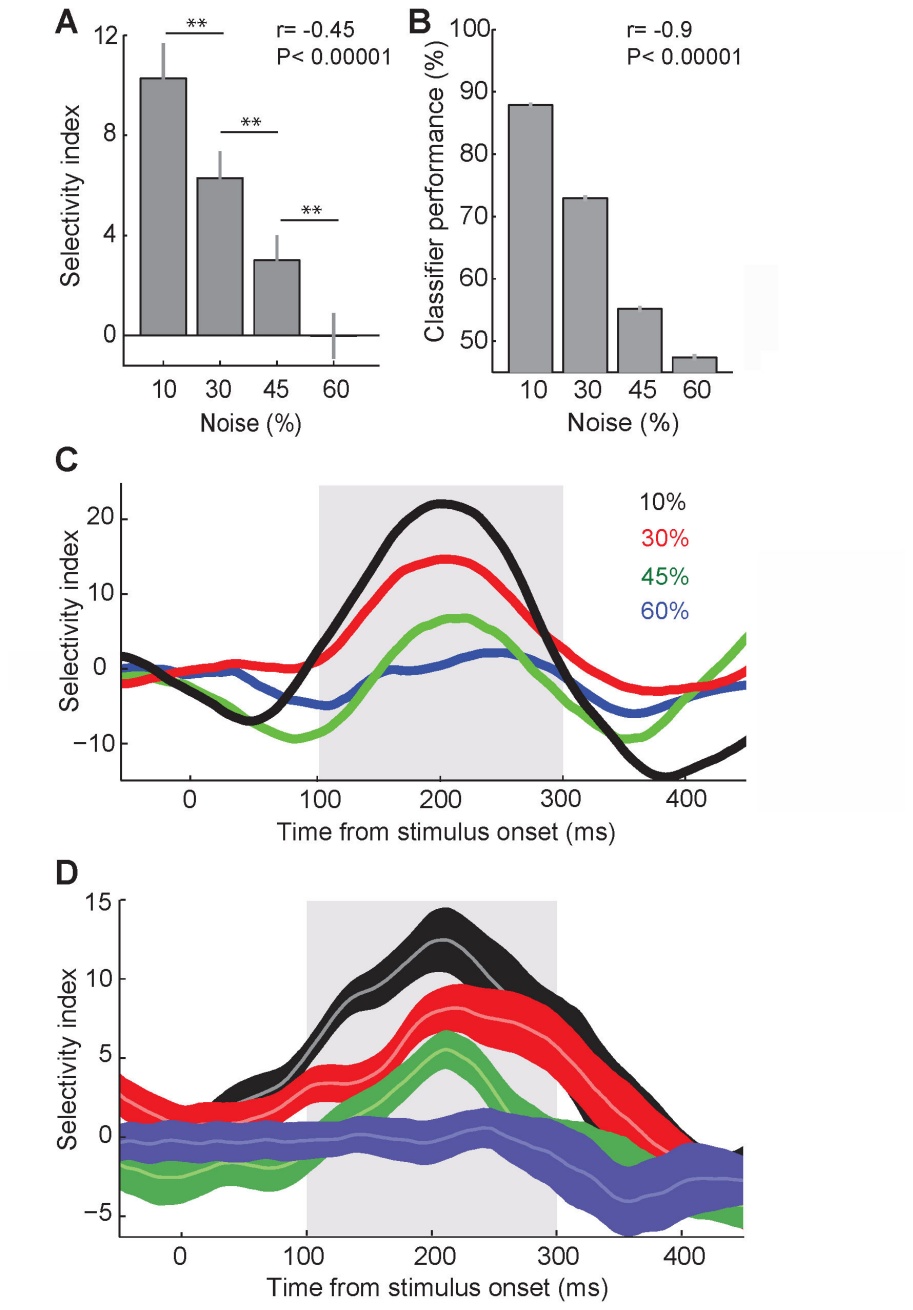
Category discriminability in different noise levels. A. SI of the all units in different noise levels, measured during 100 to 300 ms after stimulus onset. Stars show p-values of t-tests between pairs of noise levels (**: P<0.005). B. The performance of a classification trained to categorize body versus object stimuli. Stars show p-values of t-tests between pairs of noise levels (*****: P<0.00001). C. Temporal dynamic of SI of the exemplar unit (U10). SI was measured in different noise levels in sliding 50-ms windows. Data points are plotted at the middle of each bin. The gray box represents the window used for the analysis in A. D. Temporal dynamic of SI of all body selective units in different noise levels. The gray box represents the window used for the analysis in B. Conventions as in C.

To explore how the difference of the response to body and object images changes as a function of noise, we plotted the mean response to these images in different noise levels during 100 to 300 ms after stimulus onset (Figure 4C). As the noise level increased the difference of the response amplitude of the units to body and object images diminished. At 10% noise level the response amplitude to body image was significantly greater in comparison with the response to the object image. There was no statistically significant difference in response amplitude to body compared to object images at 60% noise level (t-tests, paired, one-tailed, 10%: *P*< 0.00001, 30%: *P*< 0.00001, 45%: *P*= 0.002, 60%: *P*= 0.46). Measuring SI at different noise levels in the exemplar unit (U10) showed a gradual decrease in SI as stimulus ambiguity increased (90%: 18.15, 70%: 12.87, 55%: 3.95, 40%: 0.08). Measuring SI in all units confirmed the same observation: SI of the body selective units decreased as stimulus ambiguity increased (Figure 5A, Pearson correlation; both monkeys: *r*= -0.45, *P*< 0.00001; monkey1: *r*= -0.46, *P*< 0.00001; monkey2: *r*= -0.42, *P*= 0.0006). The difference of the response amplitude to preferred and non-preferred categories decreased to the point of no difference at 60% noise level. Therefore, at this noise level SI was not significantly different from zero (t-tests, paired, *P*= 0.5) meaning that the threshold of noise tolerance for category selectivity of IT units was 60%. Note that the evoked response to both body and object images at 60% noise was significantly larger than both the baseline activity (baseline window: -50 to 0 ms relative to the stimulus onset; t-tests, paired, bodies: *P*<0.00001, objects: *P*<0.00001) and the response to full noise images (t-tests, paired, bodies: *P*<0.00001, objects: *P*<0.00001). This suggests a neural signal that indicates the presence of a stimulus but not its category; as there was not any significant difference between responses to body and object categories.

To see how responses of the population of MUs in IT could represent stimulus category in noisy conditions, we trained a classifier to categorize body versus object stimuli (Figure 5B). Classification performance decreased as the noise increased (Pearson correlation, *r*= -0.9, *P*< 0.00001). The observed chance level performance of the classifier for the stimuli with 60% noise level is a further indication that, in passive viewing condition and at high noise levels, IT units convey information about stimulus presence without signaling the stimulus category.

To explore the SI temporal dynamics we measured SI in sliding 50-ms windows at various noise levels. Figure 5C shows the results for the exemplar unit. SI in less ambiguous stimuli increased earlier and decayed later. Temporal dynamic of SI in all units, at different noise levels showed a similar pattern (Figure 5D). At 60% signal SI fluctuated around zero during the evoked response which is consistent with the lack of selectivity in Figure 5A for this noise level.

**Figure 6:**
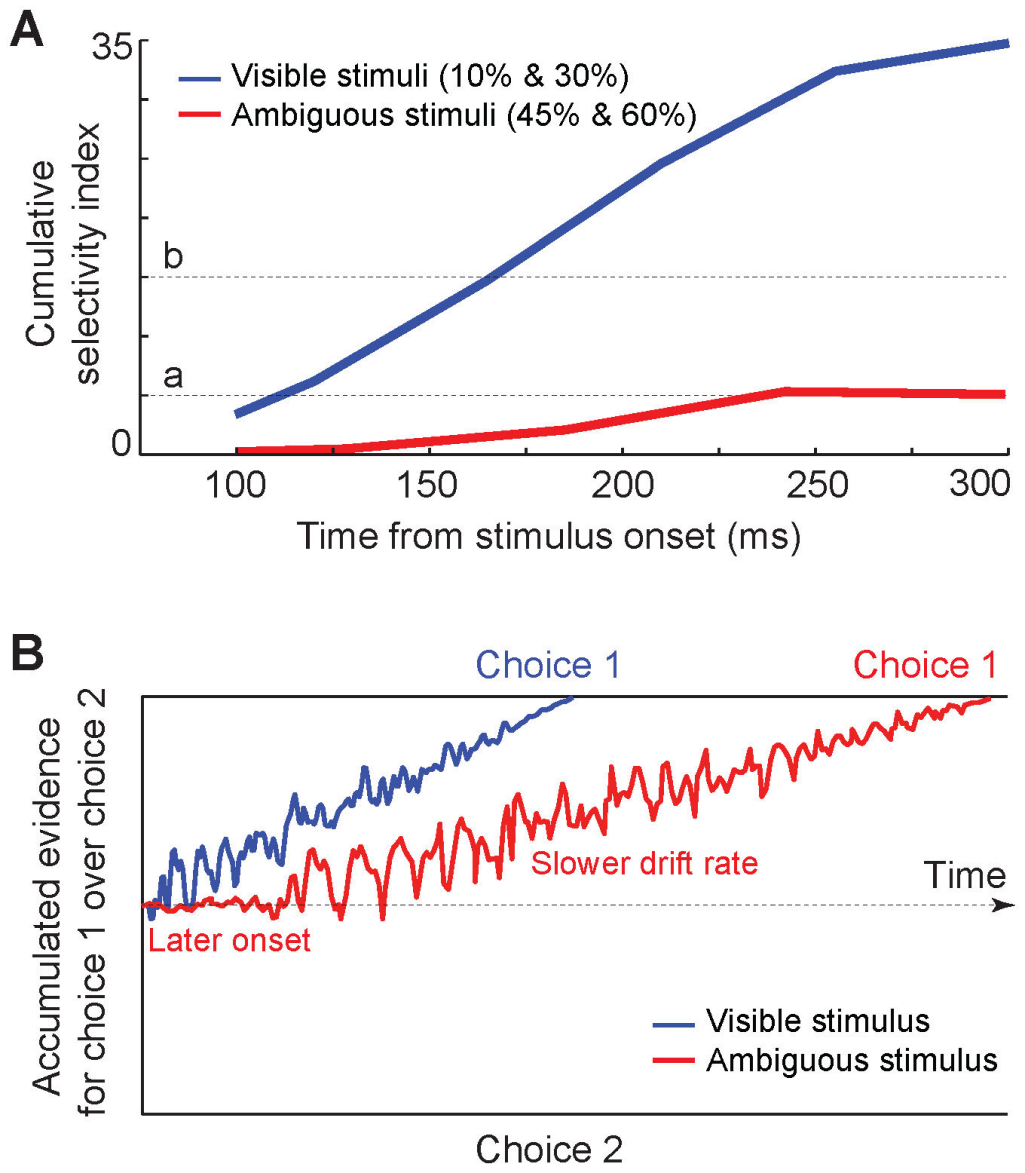
Schematic model. A. Body cells’ cumulative SI in more and less noisy conditions. Cumulative SI was measured separately for less (10% and 30%, blue line) and more (45% and 60%, red line) noisy images in non-overlapping 50-ms windows during 100 to 300 ms after the stimulus onset. Dashed lines (a and b) represent hypothetical lines representing possible decision boundaries. B. Drift diffusion model and evidence accumulation for visible and ambiguous stimuli. Decision variable (DV) is the cumulative sum of the evidence. The bounds represent the decision boundaries for different choices. Slower drift rate in ambiguous condition is the result of lower response amplitude, shorter response duration and smaller response selectivity in this condition.

We measured body cells’ cumulative SI which is the differential response to body versus object images developing in time. It was calculated separately for low (10% and 30%, blue line) and high (45% and 60%, red line) noise images during 100 to 300 ms after the stimulus onset (Figure 6A). The enhancement of SI started later and showed a shallower slope in the noisier condition.

## Discussion

To understand the neural representation of visual object ambiguity we recorded the activity of the units in IT cortex while monkeys were passively viewing body and object images with different levels of noise.

We posed four questions in the introduction to be addressed in this experiment:

1) What is the relationship between the level of stimulus ambiguity and response amplitude? We found a gradual decrease in the IT neural response to both preferred (body) and non-preferred (object) categories as ambiguity increased. Our results are consistent with previous findings showing a decrease in the IT neural responses when images are scrambled [42], morphed [40] or partially occluded [43]. fMRI studies have also shown a decrease in the response to scrambled images compared to natural images in the IT cortex of macaque monkeys [44] and Lateral Occipital Complex (LOC) of humans [45,46].

Similar to the changes in other properties of the image (size, orientation, color, viewing angle and position) IT units showed some tolerance to increased image ambiguity. The decrease in the response was gradual and even at 60% noise, preferred and non-preferred categories evoked responses which were larger than both their baseline activities and full noise.

Here we have tested the effect of noise on the neural response of IT units at a purely sensory level in a passive viewing task. The neural responses in the context of a discrimination task which is considered more demanding could have been different from the passive task in one of the following ways: First, consistent with a “response gain model” [47,48] the firing rate can be multiplied by a constant gain factor, resulting in greater enhancement of responses for the less noisy stimuli. Therefore, we could see a larger difference in the response amplitude between the high and low noise conditions. Second, in the discrimination task a constant amount of activity might be added to every response in different levels of noise. This is consistent with the “offset model” [49]. In this model the difference in the response amplitude between the high and low noise conditions would remain unchanged. Third, higher levels of task engagement in a discrimination task could make neurons more sensitive to the noisier stimuli with less visual information. Thus, similar to the “contrast gain model” [50-52], task demand and visual signal act interchangeably. Therefore, we could see a smaller difference in the response amplitude between the high and low noise conditions. These possibilities remain to be tested by comparing the neural responses to ambiguous stimuli in a passive viewing and a body/object discrimination task.

2) What is the effect of noise on the temporal dynamic of the neural responses? Our results showed the occurrence of a later response onset, earlier response offset and shorter response duration as the level of ambiguity increased. Here, for the first time, we demonstrate the effect of stimulus ambiguity on the temporal pattern of responses in visual cortex. It is important to note that the evoked response amplitude by itself does not give such information about the temporal pattern of the response. Responses with different amplitudes might have similar onset, offset or duration and vice versa. In one of our previous works we have presented the PSTH of an exemplar face selective cell in the IT cortex with shorter onset latency, larger amplitude and shorter duration of the response to human faces compared to animal faces [41]. In the same study we have shown that in the population of IT cells while the response amplitude for human faces and animal faces are similar, the response onset latency for human faces in significantly shorter than animal faces.

It has been shown that the IT neurons respond to illusory contours [53-55]. Illusory-border defined shapes induce longer response onset latencies compared to their counterpart real images. The longer response latency for illusory contours compared to real contours suggests the possibility of analyzing the visual information within IT columns [4] or top-down feedback for processing of subjective contours [56,57]. It is likely that longer response onset in noisier conditions in our task is related to similar processing mechanisms to retrieve the lost information in the images.

3) What is the relationship between the category selectivity and various levels of noise? Our results regarding the response amplitude showed that the neural response in full noise (100% noise) was larger than baseline activity. Although there is no meaningful visual information in fully noisy images, they could evoke IT units. This suggests that while amplitude of spiking activity is an important measure in the neural coding, it is not enough for understanding the neural basis of object representation in IT.

Category selectivity is one of the intriguing properties of IT units. Some studies have shown that category information is represented in the neural activity of IT cortex [2,42,58-60]. Previous work from our laboratory has demonstrated the causal link between category selective units in IT and visual categorization performance [5]. The same study has shown that IT units with larger category selectivity contribute more to the behavior. We examined how this critical property of IT units is affected by noise and found that category selectivity gradually decreased as the noise level increased. This result is consistent with previous findings in area MT of dorsal visual pathway [39]. We found that category selectivity was lost in 60% noise which means that the threshold of noise tolerance for IT category selectivity was 60% in our task. At this noise level, the preferred and non-preferred categories evoked similar responses which were larger than both their baseline activities and full noise. These findings suggest that discrimination requires a larger signal to noise level than detection. Such a condition occurs when we see a noisy visual stimulus and we know there is something out there but we do not know what it is [61-63]. We have to note that while individual MU responses examined in our study may fail to convey category information at high noise levels (60%) a larger population of neurons could still signal category information at such a noisy condition since the neural sparsity might potentially decline as stimulus noise increase [64]. However, our finding that, in passive viewing condition, the classifier performed near chance level for the stimuli with 60% noise suggests that the neural system may indicate the presence of visual object without signaling what the object is.

4) What is the neural mechanism of decreased accuracy and speed in recognizing more ambiguous stimuli? Psychophysical studies have found that the accuracy and speed of object recognition and the amount of visual noise are inversely correlated. Here, by explaining our findings in the context of drift diffusion model (DDM), we introduce the underlying mechanisms of these behavioral findings. The DDM has received increased attention over the past few years for providing a better description of accuracy and reaction time of making a decision compared to alternative models [65-68]. In this model the decision variable (DV) is a cumulative sum of the evidence. When DV reaches one of the stop bounds the decision is made (Figure 6A).

Previous studies have shown that lesions of the temporal lobe cause deficits in the object recognition performance [69]. We also know that electrical stimulation of the temporal lobe induces the imagery recall in humans [70]. We have reported that microstimulation of the category selective neural clusters in IT modulates the object categorization performance [5]. The same study showed that IT clusters with larger category selectivity contribute more to the behavior. The selectivity of IT neurons to complex objects such as bodies and the effects of lesions and microstimulation of the IT cortex on the object recognition performance indicate a crucial role for this area in perceptual categorization. It has also been shown that the choice signal is present in the IT neural responses in the context of depth discrimination [71] and visual search tasks [72]. These studies suggest that IT neural activity can be a manifestation of the decision variable evolving in this area.

Based on DDM model, the speed of making a decision depends on the start point of the accumulator and the drift rate. The start point corresponds to the neural baseline activity [73]. Our task was not block designed for different levels of ambiguity. Hence, the monkey had no clue what stimulus would be presented in the upcoming trial, which makes the pre-stimulus condition exactly the same for all of the trials. Drift rate or the slope of the DV trajectory moving toward the decision boundaries depends on the rate of evidence accumulation. In our results the response onset latency was longer in noisier conditions. Therefore, evidence accumulation and the drift started later in these conditions. Furthermore, the response amplitude and selectivity were smaller which provided less information at any given time. This could result in a slower drift rate in noisy conditions. Due to the later start of the drift and the slower drift rate, it takes longer for the DV to reach the decision boundary in noisier condition. Figure 6B represents a schematic illustration of this mechanism which explains longer reaction times observed in behavioral studies. Cumulative SI presented in Figure 6A could be an indicator of the DV accumulation across time. In the noisier conditions, later onset of the drift and also the slower drift rate was associated with a slower accumulation of the DV compared to the less noisy conditions.

Slow accumulation of the DV makes it reach the boundary later (e.g. line ‘a’ in Figure 6B) or do not reach the boundary at the time of decision making (e.g. line ‘b’ in Figure 6B). The accuracy of making a choice depends on how close DV is to the decision boundary at the time of making a choice. Later onset, smaller amplitude, decreased selectivity and shorter duration of response in noisier conditions decrease the available evidence for formation of DV. Therefore, at any given time the accumulated evidence for noisier conditions is farther from the decision boundary which makes the choices less accurate. In such a condition the trade-off between accuracy and speed might help the subject to accumulate more evidence in a longer time to make a more accurate decision.

## Conclusions

By measuring the neural response of the IT cortex we found that an increase in the level of ambiguity of visual objects gradually decreases the amplitude and selectivity of the response. In terms of temporal dynamic of the response, stimulus ambiguity gradually increases the onset latency and decreases the offset latency and duration of the evoked response. We explained the possible mechanisms underlying of the changes in the accuracy and speed of object recognition by a drift diffusion model of decision making. We believe that our findings are important for a better understanding of the neural basis of object recognition in ambiguous conditions and also the mechanisms of behavioral changes in such situations.

## Supporting Information

Figure S1 **Image set.** The stimuli were grayscale photographs of bodies (humans, monkeys and quadrupeds) and objects (aircraft, car and chair). There were 30 images per subcategory (90 images in each category). Each stimulus was presented in four different noise levels (10, 30, 45 and 60 percent).
(TIF)
