# Supplementary figures and images for: Neural Representation of Ambiguous Visual Objects in the Inferior Temporal Cortex
Source: PLoS One. 2013 Oct 3;8(10):e76856. doi: 10.1371/journal.pone.0076856 (PMC3789700; doi:10.1371/journal.pone.0076856)

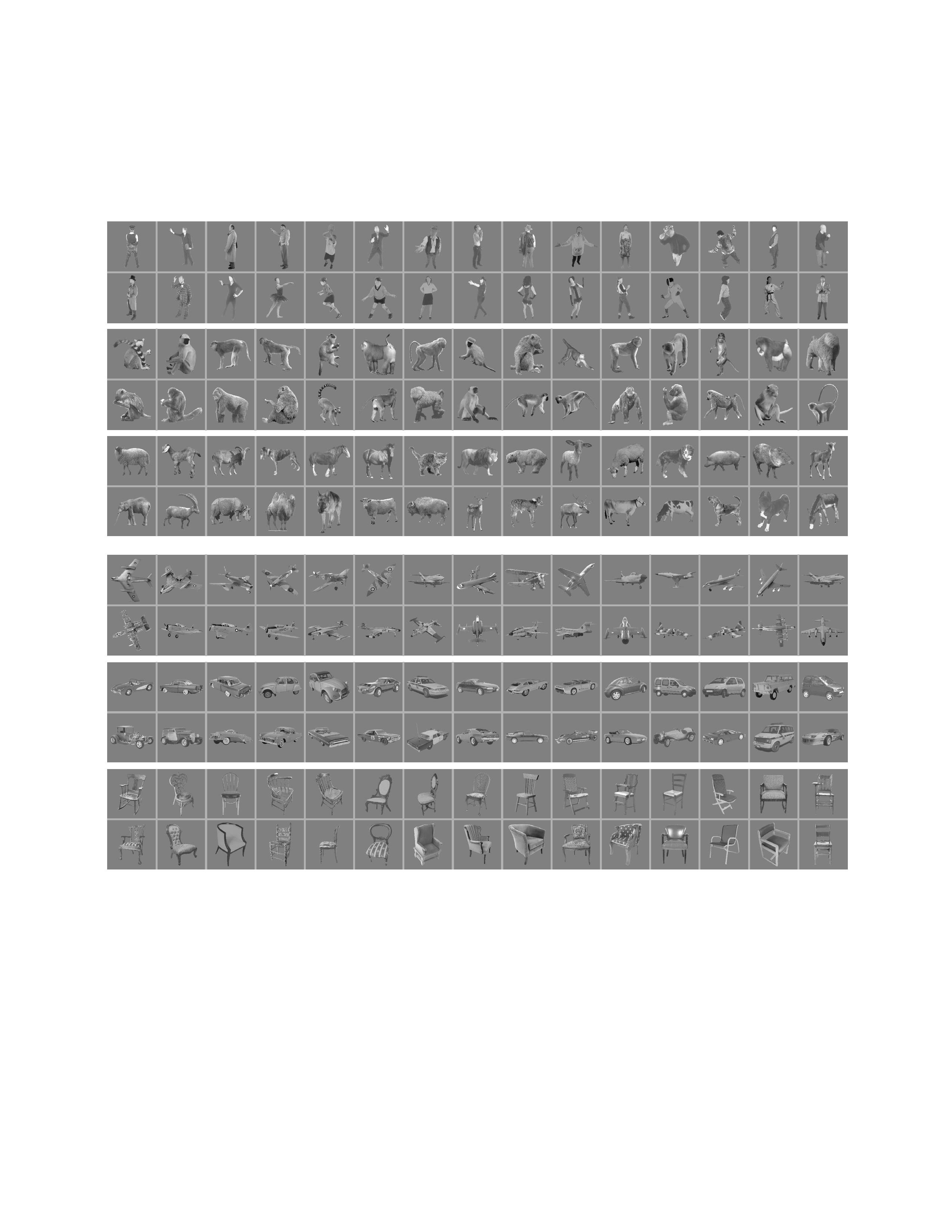

Supplement: Figure S1 — Image set. The stimuli were grayscale photographs of bodies (humans, monkeys and quadrupeds) and objects (aircraft, car and chair). There were 30 images per subcategory (90 images in each category). Each stimulus was presented in four different noise levels (10, 30, 45 and 60 percent). (TIF) [file pone.0076856.s001.tif]
